# Reasons why OCT Global Circumpapillary Retinal Nerve Fiber Layer Thickness is a Poor Measure of Glaucomatous Progression

**DOI:** 10.1167/tvst.9.11.22

**Published:** 2020-10-19

**Authors:** Melvi D. Eguia, Emmanouil Tsamis, Zane Z. Zemborain, Ashley Sun, Joseph Percival, C. Gustavo De Moraes, Robert Ritch, Donald C. Hood

**Affiliations:** 1 Einhorn Clinical Research Center, New York Eye and Ear Infirmary of Mount Sinai, New York, NY, USA; 2 Department of Psychology, Columbia University, New York, NY, USA; 3 Department of Ophthalmology, Columbia University Irving Medical Center, New York, NY, USA

**Keywords:** glaucoma, optical coherence tomography, progression, summary metrics

## Abstract

**Purpose:**

To assess the effects of local defects, segmentation errors, and improper image alignment on the performance of the commonly used optical coherence tomography (OCT) measure of progression, that is the change in global (average) circumpapillary retinal nerve fiber layer (cpRNFL) thickness (ΔG).

**Methods:**

One hundred fifty eyes suspected of, or with, early glaucoma had OCT circle and cube scans obtained using eye tracking on two occasions at least 1 year apart. Statistical progression was defined by fixed values of ΔG (3–8 um) and quantile regression. For a reference standard, four authors identified 30 eyes as “likely progressed,” and 61 eyes that “likely had not progressed” based on OCT reports from both baseline and follow-up tests.

**Results:**

A ΔG criterion of 4 um had the best accuracy: 77%, with 5 false positive (8.2%) and 16 false negative (53%). A post hoc analysis of circular b-scans and OCT probability maps of these eyes indicated that segmentation errors and local progression accounted for most of these mistakes. Segmentation errors, although less common, were also present in true positives and true negatives.

**Conclusions:**

Local defects and segmentation errors are the primary reasons for the poor performance of cpRNFL thickness G metric. Because these problems are difficult, if not impossible, to eliminate, the G metric should not be relied on in isolation for detecting glaucomatous progression.

**Translational Relevance:**

Local defects and segmentation errors are easily identified by viewing OCT circumpapillary images, which should be part of the standard protocol for detecting glaucomatous progression.

## Introduction

Identifying glaucomatous progression is key to the clinical management of patients with glaucoma. The general consensus is that optical coherence tomography (OCT) can, and should be, employed in ascertaining if a patient with glaucoma is progressing. However, there is less agreement about how best to detect progression with OCT.

One common approach for detection of progression employs the global average thickness (G) of the circumpapillary retinal nerve fiber layer (cpRNFL) obtained for an OCT circle scan around the disc.^1^ Summary statistics such as G have traditionally been developed to scale down the plethora of information and to reduce variability. Previous studies have defined the confidence limits of test-retest variability for a change in G (ΔG) to be slightly below 5 µm, based on repeated testing in healthy patients conducted over a short interval.^2^^–^^4^ These limits of variability led to an informal “rule of 5,” which has been used by clinicians for detecting glaucoma progression.^2^ Based on this rule, when an eye shows a loss of 5 µm or more in ΔG, this is considered quantitative evidence of glaucomatous progression. However, recent work suggests that this “rule of 5” has poor specificity (i.e., it leads to too many false positives [FPs] over time).^5^^,^^6^

A recent study by our group,^7^ suggested there are at least three reasons for the poor performance of the ΔG metric. One, it can miss local defects, including those responsible for arcuate damage near fixation. Two, relatively subtle errors in segmentation can produce ΔG values of at least 3 to 4 um, as can errors in alignment, such as differences in the centering of the disc between sessions.^8^ As is typical for the most commonly used OCT instruments, that study derived the circle scan image from a single cube scan and aligned the scans from different days based on software determination of the disc center.^9^

Because the ΔG is commonly used, it is important to better understand the problems associated with it, as well as to assess the extent to which these problems can be mitigated. In this study, we test a different group of patients with early glaucoma and glaucoma suspects, using a different OCT instrument and a different protocol. In particular, the protocol included averaged circle scans to produce circumpapillary b-scan images. The resulting images have better resolution than those available from derived circle images. Thus errors in segmentation and alignment are easier to visualize and assess. Further, the instrument uses eye tracking to place the follow-up scan in the “same” location in relation to the center of the disc.^10^ Thus we test the hypothesis that local defects, segmentation errors, and alignment play a role in negatively affecting the performance of the commonly used change in the cpRNFL thickness, the ΔG metric. We predict that local defects and segmentation error will negatively impact performance of the G metric, but that the eye tracking may minimize alignment errors.

## Methods

### Participants

The study group consisted of 150 eyes from 96 patients referred for OCT imaging by one of the authors (RR). According to the referring physician, the eyes were suspected of glaucoma or had early glaucomatous damage. All eyes had a visual field 24-2 mean deviation better than −6 dB. Of the 150 study eyes, 25 (16.7%) had a refractive error lower than −6 diopters (D) and could therefore be characterized as high myopes. The median refractive error of this subset was –7 D (interquartile range, 3.1; range, –6 to –15). We chose not to exclude these high myopic eyes so as to maximize clinical relevance.

Each eye was required to have at least one reliable visual field test performed using the Swedish Interactive Threshold Algorithm (SITA) standard 24-2 testing strategy on a Humphrey Field Analyzer II-I (Carl Zeiss Meditect, Inc., Dublin, CA). A visual field was defined as being unreliable if there were greater than 15% FP errors or greater than 33% fixation losses or false-negative (FN) errors. All eyes were scanned at least twice: one baseline (“baseline”) scan and another (“follow-up”) at least 1 year after the baseline scan. Eyes were excluded if they had any ocular or systemic conditions that could affect visual field or OCT imaging results (e.g., retinal vein occlusion, demyelinating disease). Any scan affected by significant blink or eye movement artifacts was also excluded.

The institutional review boards of Columbia University and the New York Eye and Ear Infirmary of Mount Sinai approved this study, which adhered to the tenets of the Declaration of Helsinki and the Health Insurance Portability and Accountability Act. Written informed consent was obtained from all participants.

### OCT Imaging

All eyes were scanned with the Spectralis HRA+OCT with the Glaucoma Module Premium Edition (GMPE) protocol (Heidelberg Engineering, Inc., Heidelberg, Germany), which acquires circle and cube scans using eye tracking to help place the scan in the same location at follow-up sessions. All cube scans are obtained along the fovea-to-Bruch's membrane opening center axis.

**Figure 1.**
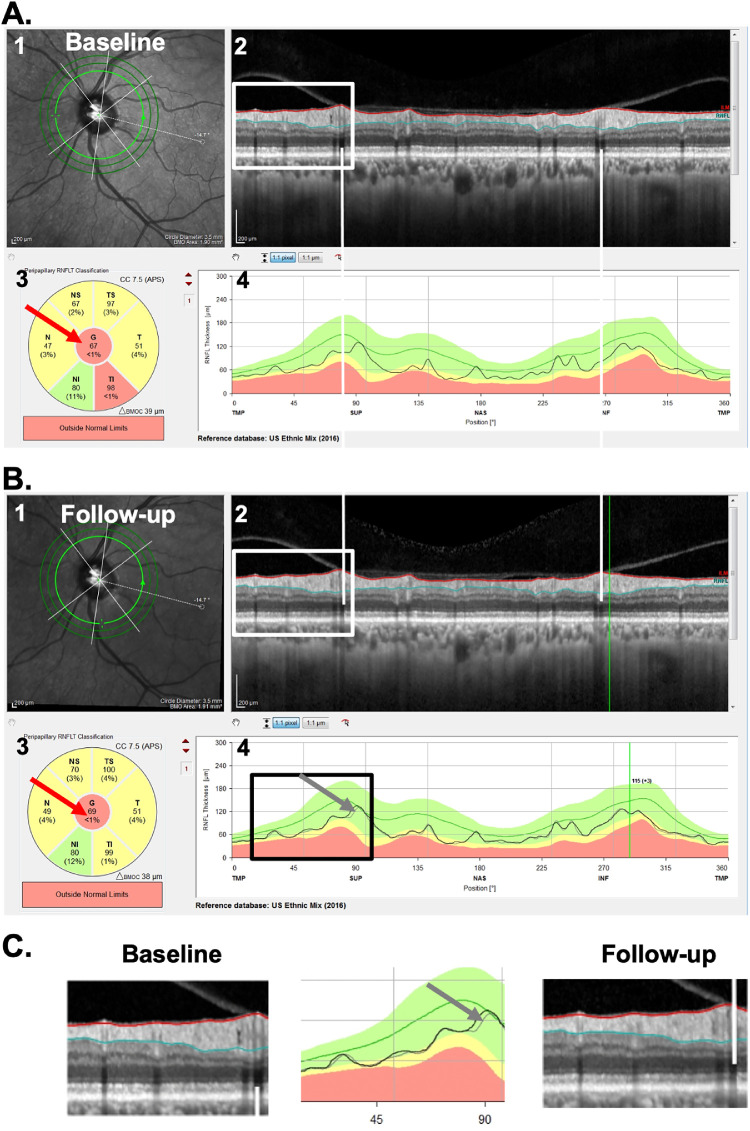
(A) The commercial circle scan report for a baseline test of a patient (ID46) showing the (1) infrared image of the disc, (2) averaged circular b-scan image, (3) cpRNFL thickness pie chart, with *red arrow* pointing to the global metric (G), and the (4) cpRNFL thickness profile. (B) The follow-up report for the same eye. (C) Enlarged images of the portion of panels A and B within the *white* and *black* rectangles. In the center panel, the baseline curve is in *gray* (pointed by a *gray arrow*) and the follow-up is in *black*.

Fundamental to this study is the cpRNFL report in Figure 1A. This commercial report is based on the 3.5-mm diameter circle scan of the GMPE protocol. It shows a large image of the circumpapillary b-scan (panel 2) and a plot of the cpRNFL thickness around the optic disk as the black curve in panel 4. On the report for the follow-up visit (Fig. 1B), the thickness curve from the baseline visit is shown as a gray curve (gray arrows in Figs. 1B, 1C) for direct comparison with the thickness (black) curve of the current/follow-up scan. In addition, the report shows, the average cpRNFL thickness for regions of the circle scan, including the overall global average, G, which is in the center of the pie charts (red arrows, Fig. 1).

### Definition of Statistical Progression

Two standard techniques were used to define statistical progression. First, we set the cutoff for progression based on fixed values of ΔG. In particular, progression was evaluated for a cutoff loss of 5 um (the “rule of 5”),^5^ as well as 3, 4, 6, 7, and 8 um. In addition, we applied quantile regression (QR), using a previously described group of patients.^11^ In brief, baseline and short-term (<6 months) follow-up circle scans were acquired from participants in the Macular Damage in Early Glaucoma and Progression trial (ClinicalTrials.gov identifier: NCT02547740), with the same imaging (i.e., GMPE) protocol. The baseline G values were set as the independent variable, whereas all follow-up G values were the dependent variable. The 95th percentile defined the criterion/cutoff for “statistical progressors.” (Note: this approach is analogous to that employed by commercially available visual field and OCT machines in their glaucoma progression analyses.) These cutoffs were then applied on our study group. They varied from −1.4 to −4 um, depending on the baseline G thickness.

### Reference Standard (RS) and Post Hoc Analysis

Our purpose here was to better understand the problems associated with using ΔG to identify progression, not to assess or determine the G metric's sensitivity and/or specificity. In particular, our approach depended on identifying eyes that we were reasonably confident had either likely progressed (P) or eyes that likely had not progressed (NP). This was followed by a post hoc analysis, described later.

**Figure 2.**
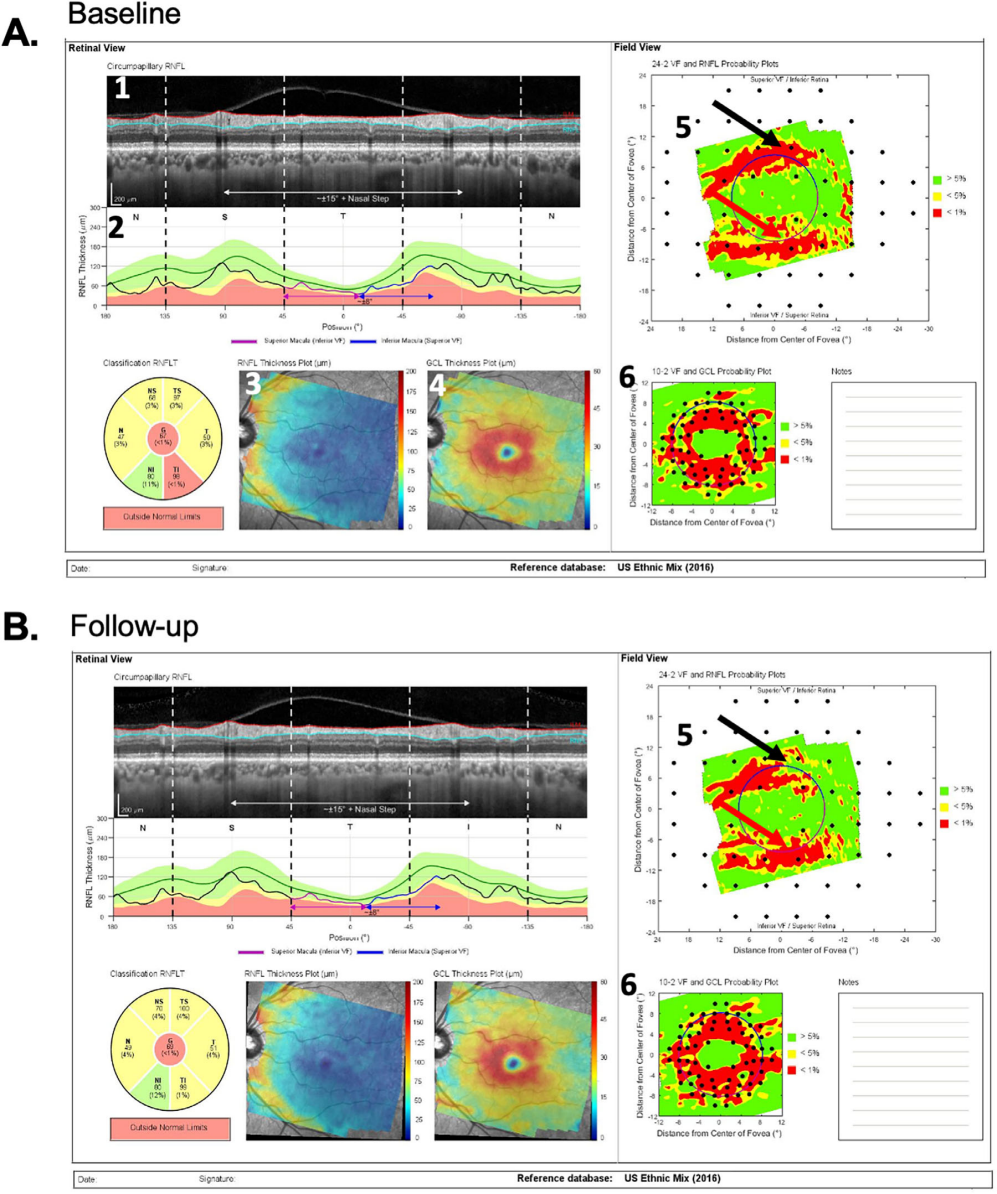
(A) The prototype of a commercial report for a baseline test of the same patient as in Figure 1 with the (1) cpRNFL circular b-scan, (2) cpRNFL thickness profile, (3) RNFL thickness map, (4) retinal GCL thickness map, (5) field view of the RNFL probability map, and (6) field view of the GCL probability map. (B) Same as (A) for the follow-up test.

To determine which of the 150 eyes were P and which were NP, four of the authors (MDE, ET, AS, and DH) independently evaluated the OCT reports from both baseline and follow-up tests, and judged whether each eye had progressed on a scale of 0 (definitely did not process) to 100% (definitely did progress); differences were adjudicated, and consensus was reached, through a collective qualitative evaluation of the baseline and follow-up reports, and careful inspection of the circumpapillary b-scans and their corresponding cpRNFL thickness plots (see Fig. 1). Progression was confirmed with the retinal nerve fiber layer (RNFL) and ganglion cell layer (GCL) deviation/probability maps (see Fig. 2). Those eyes with scores of 95% or more were categorized as P, and those with scores of 5% or less as NP.

Figures 1 and 2 show examples of the two reports evaluated by the OCT experts. One report was the commercially available circle scan report in Figure 1 described earlier; the other was our laboratory-based, one-page report described in our previous work,^9^^,^^12^^,^^13^ and shown in Figure 2. The report in Figure 2 is based on both the 3.5-mm circle scan and the 61 horizontal line cube scan of the GMPE protocol. It includes a cpRNFL b-scan image (1) from the optic disc circle scan, and its corresponding cpRNFL thickness profile (2). Both the b-scan and the thickness profile were presented with the temporal region of the disc at the center to provide greater ease for evaluating the topographic relationship between the cpRNFL thickness profile and retinal and visual field locations. This report also includes the RNFL (3) and GCL (4) thickness maps. Corresponding probability plots for each of these thickness plots are also included (5 and 6). These are presented in field view, that is, with the inferior retina/superior visual field on top. Parts of this report are already incorporated into the commercial Heidelberg software, and other parts (probability maps) are under research development and for investigational use only.

Figures 1 and 2 show the reports for the baseline (A) and follow-up (B) tests of an NP eye. First, the cpRNFL thickness curves for the baseline and follow-up tests were very similar, as indicated by the overlap of the gray and black curves in panel 4 of Figure 1B. In this particular example, there are small deviations between the two curves, that resulted in a positive ΔG value of 2 um. Second, the probability maps in Figures 2A and 2B were also similar. For example, the black and red arrows in Figures 2A and 2B show abnormal regions of similar size. Taken together, the near identical cpRNFL thickness curves combined with similar RNFL and GCL maps resulted in a judgement of “likely not progressing” (NP).

**Figure 3.**
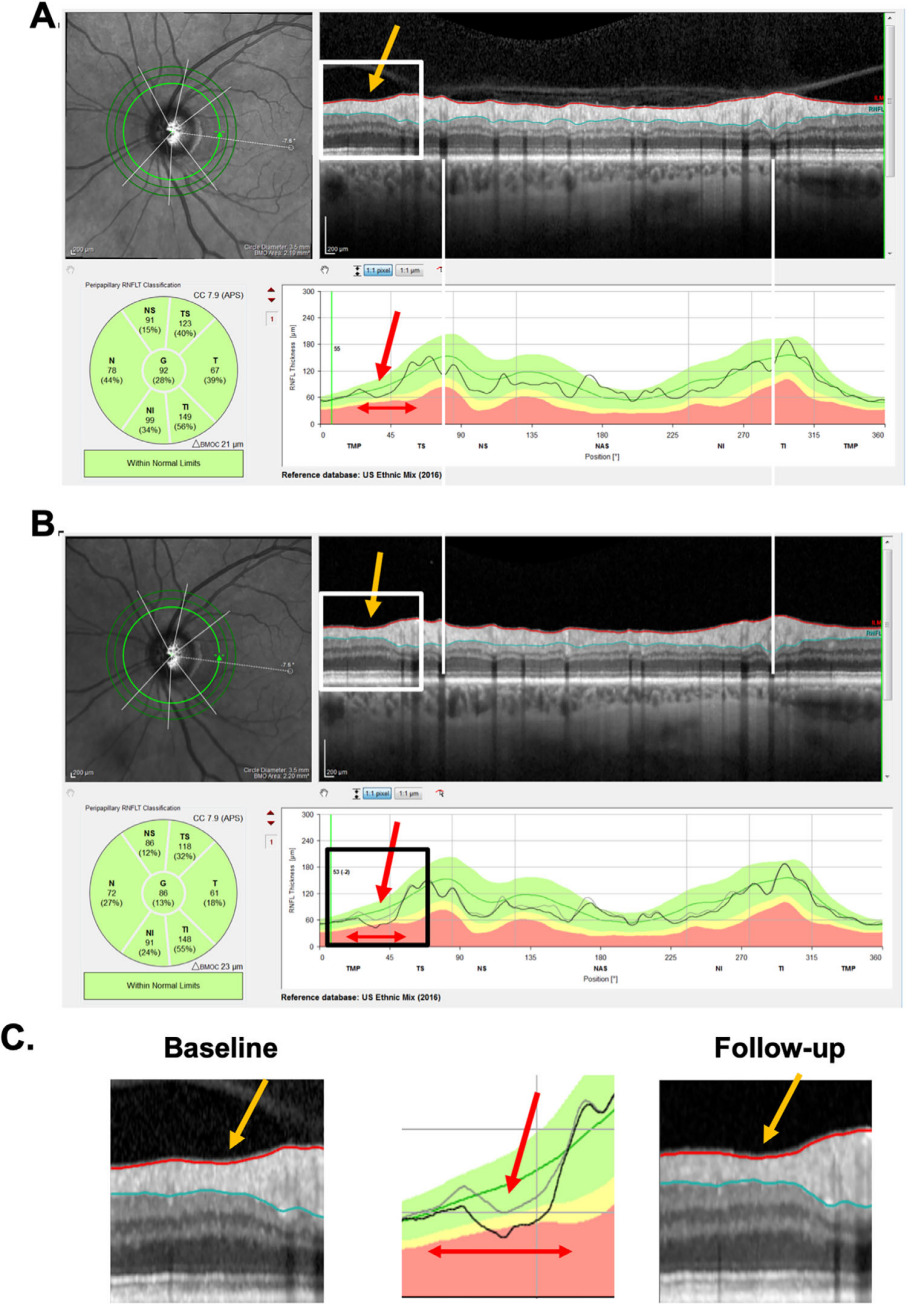
(A–C) Same as in Figure 1 for patient ID147. A local region of progression is denoted by the *orange arrow* in the b-scan and the *red arrow* in the thickness map, and its width by the *double-ended red arrow*.

**Figure 4.**
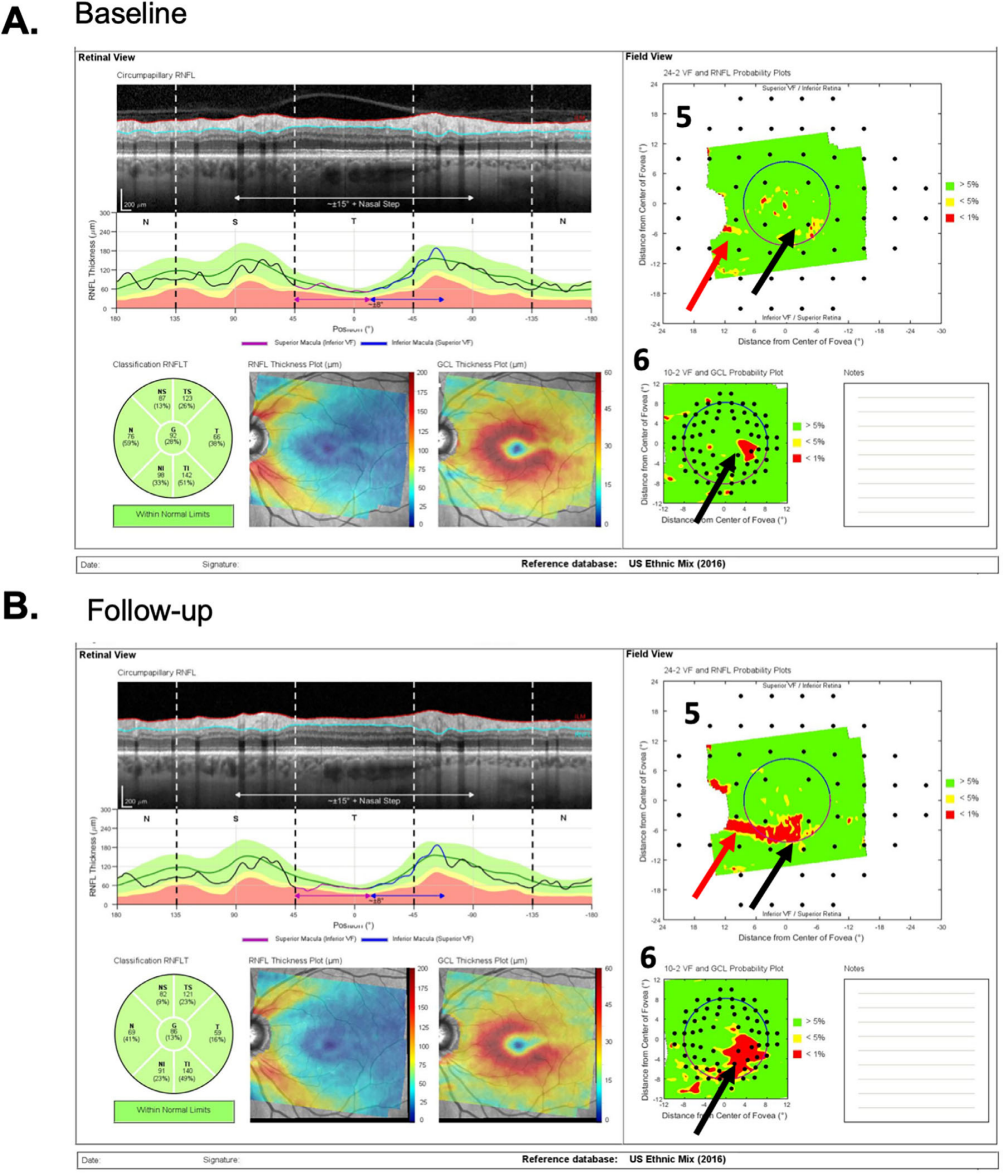
(A and B) Same as in Figure 2 for patient ID147. “Likely progression” was confirmed on the probability maps (5 and 6), where the *red arrows* indicate the same location as the local defect on the circle scan in Figure 3, and the *black arrows* topographically corresponding locations showing progressive changes.

Figures 3 and 4 show the reports for the baseline (A) and follow-up (B) tests of a “likely progressing” (P) eye. Eyes were classified as P if (1) there were regions of the cpRNFL curves that were lower on the second test (red arrows in Figs. 3A–C); (2) this difference in cpRNFL between days was confirmed on the b-scan (orange arrows in Figs. 3A–C); and (3) the change over time was confirmed on the probability maps in Figures 4A and 4B in the same (red arrows) and topographically corresponding (black arrows) locations.

### Post Hoc Analysis

Based on this classification of NP and P, we determined FPs and FNs and performed a post hoc analysis to better understand possible problems with the G metric for progression. For this, we evaluated all of the OCT circle scan images and thickness plots (e.g., Figs. 1A [panel 2], 1A [panel 4], 1B [panel 2], and 1B [panel 4]) to detect factors that may affect ΔG values. In particular, we assessed the role of three factors that prior work^7^ suggested could contribute to FP and FN based on ΔG: (1) the presence of local defects, (2) alignment errors, and (3) segmentation errors.

A local defect was defined as an arcuate RNFL defect visible on the RNFL probability plot (e.g., arrows in Fig. 4) that is topographically associated with a local depression of RNFL thickness on the cpRNFL thickness plot and circular b-scan (arrows in Fig. 3). On the cpRNFL plot (Fig. 3), to be considered local, the associated region had to be less than 45° wide. A wide-spread defect, however, was defined as one for which the cpRNFL curve fell in the yellow or red abnormal range for at least 90°.

Alignment was evaluated based on the location of shadows from the superior and inferior temporal blood vessels corresponding to the superior and inferior region of the cpRNFL of the baseline and follow-up circle b-scans. For example, in Figures 1 and 3, the white vertical lines were placed on the shadows of the blood vessels seen on the baseline scan. A follow-up scan was deemed misaligned if the same blood vessel was shifted by more than the width of a blood vessel.

**Figure 5.**
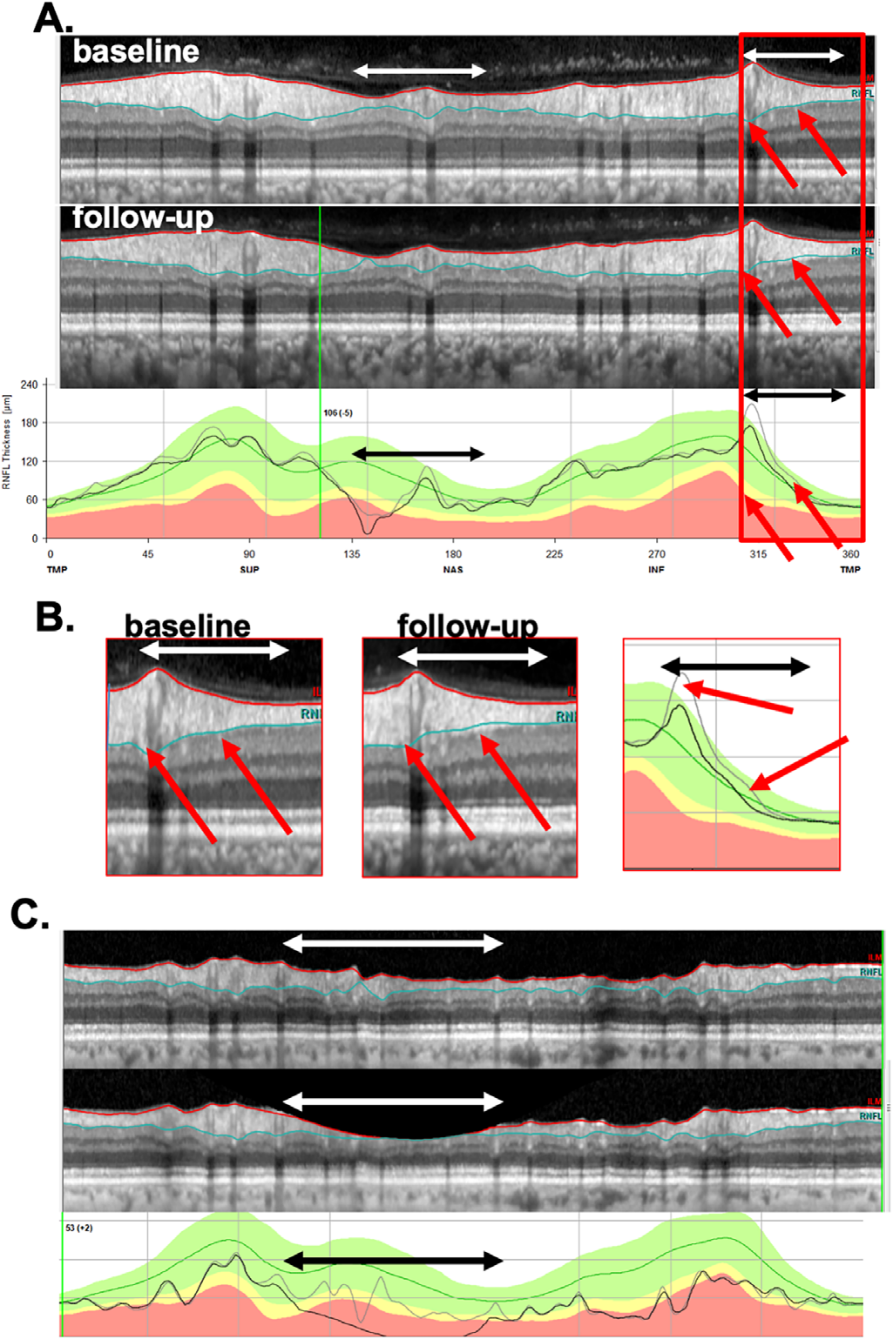
(A) Circle scan images of baseline test (*top*) and follow-up test (*middle*), along with the cpRNFL thickness profile (*lower*) for patient ID122. Regions with segmentation errors are indicated by the *double-ended white and black horizontal arrows*. (B) Enlarged images of the portion of the panels in A and B with the *red rectangle*. The *red arrows* show corresponding locations with segmentation errors. In the center panel, the baseline curve is in *gray* and the follow-up is in *black*. (C) Same as in (A) for patient ID142, where the image on follow-up shows a clipping error and the *white and black horizontal arrows* show the region affected by this error.

Segmentation was assessed in each circle b-scan by inspecting the red and blue segmentation lines that demarcate the internal limiting membraneand RNFL boundaries, respectively. A scan was considered poorly segmented if (1) the segmentation lines clearly failed to identify their borders, and (2) the poorly segmented region was larger than 5°. Figure 5 shows examples of segmentation errors.

We identified another factor that we hypothesized might affect ΔG values. In particular, in a few eyes, there was a vertical “apparent scaling” of the image that causes an apparent change in the thickness of all retinal layers, including the RNFL. We called this artifact “apparent change in retinal thickness” (aΔRT). This change in thickness between two scans can be detected if one flickers between the two images in a manner similar to flicker chronoscopy (Supplementary Video S1). In addition, we confirmed the presence of this aΔRT artifact by placing the scan images from two test dates next to each other, as shown in Supplementary Figure S1.

## Results

**Figure 6.**
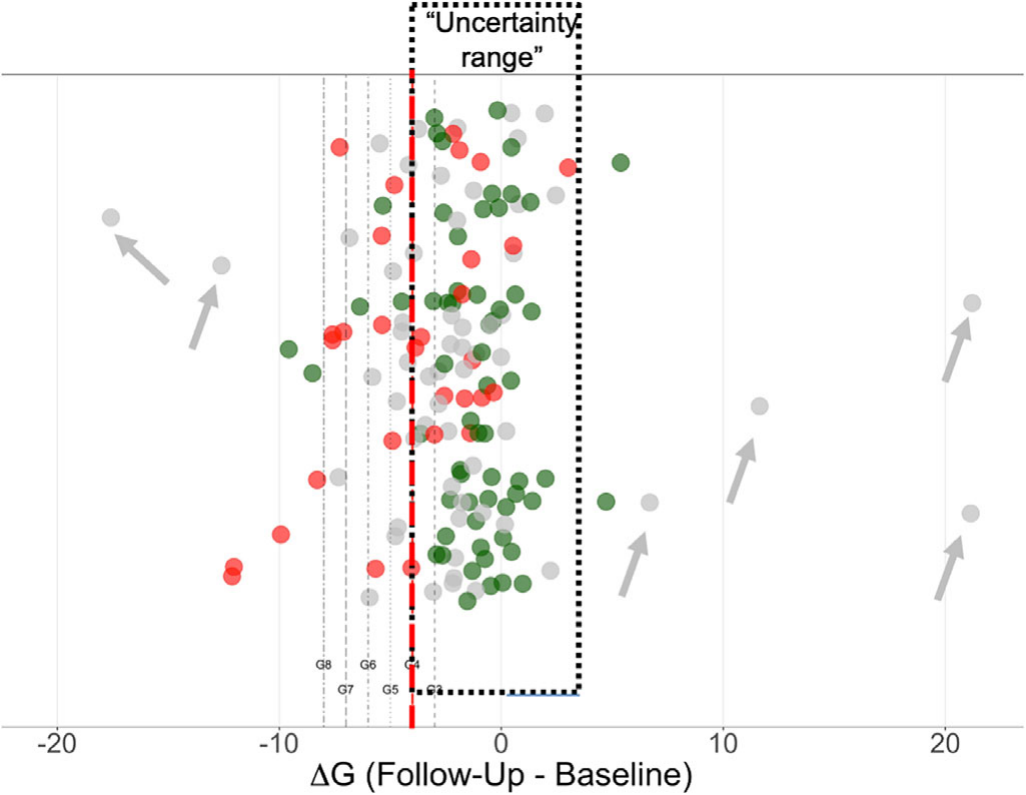
The ΔG value (change in global cpRNFL thickness [follow-up – baseline]) for each of the 150 eyes. The x-axis represents the ΔG value, with the eyes displaced along the y-axis for visualization purposes. The *vertical dashed lines* represent the ΔG criteria of −3 to −8 um. The *red circles* are the eyes in the P group based on the RS; the *green circles*, the eyes in the NP group; and the *gray circles*, the eyes in the “uncertain” group, that is, neither P or NP. The *gray arrows* indicate the outliers in the uncertain group with the extreme ΔG value.

The change in the G metric (ΔG) for each of the 150 eyes is shown in Figure 6, in which each circle represents one of the 150 eyes. The x-axis indicates the ΔG value, and the eyes are displaced along the y-axis to aid in the identification of individual eyes. There were 30 P eyes (red circles) and 61 NP eyes (green eyes) and the remaining 59 eyes (gray) were “uncertain”: neither P nor NP. The median (interquartile range, range) ΔG for P and NP was −3.8 (5.2, −12.1 to 3.0) um and −0.8 (2.6, −9.5 to 5.3) um, respectively. Pairwise comparisons using Wilcoxon rank-sum test revealed significant differences between the three groups (*P* < 0.001 for P vs. NP, *P* < 0.03 for uncertain vs. P, and uncertain vs. NP). Although the groups have significantly different ΔG values, the clinician needs to make decisions about individual eyes.

As described earlier, two methods were used to statistically categorize individual eyes as P or NP. In particular, they were categorized based on (1) fixed ΔG values, and (2) ΔG values defined by QR. The former criteria are indicated by the vertical dashed lines in Figure 6, which are associated with ΔG values of −3 to −8 um. The QR values ranged from −1.4 to −4 um. To identify eyes for a post hoc evaluation, the “performance” of the statistical criteria was evaluated against the 91 eyes classified as P or NP. Table 1 summarizes the results. None of the criteria had an accuracy better than 77%, nor a sensitivity better than 53%. Marginally, the best accuracy was for a loss of 4 um (ΔG = −4 um), the vertical dashed red line in Figure 6. Although more negative ΔG criteria (−5 to −8 um) showed 1 to 3 fewer FPs, they had 3 to 10 more FN (misses). However, QR had fewer FN, but more FP; even for the QR criterion, with the fewest FN, the FN rate was 47%.

**Table 1.** Number/Percentage of FPs, FNs, and Accuracy (%), Based on a Quantile Regression Criterion and a Set of Cutoff Loss from 5 to 8 µm as ΔG Criteria.

Criterion	FP NP = 61	FN P = 30	Accuracy (%)
QR^a^	11 (18.0%)	14 (46.7%)	73%
3	7 (11.5%)	15 (50.0%)	76%
4	5 (8.2%)	16 (53.3%)	77%
5	4 (6.6%)	19 (63.3%)	75%
6	3 (4.9%)	22 (73.3%)	72%
7	2 (3.2%)	22 (73.3%)	74%
8	2 (3.2%)	26 (86.7%)	69%

^a^Range −1.4 to −4.

### Post Hoc Analysis of FP and FN

The purpose of categorizing eyes as FP and FN was to identify eyes for our post hoc analysis. To this end, we chose the criterion with the best accuracy, −4 um. However, none of the conclusions about the factors affecting ΔG would change if the cutoffs were based on QR or a different ΔG value (e.g., −5 um).

*Analysis for FP:* For the −4 um criterion, 5 (8%) of the 61 NP eyes were FP (Table 1); in Figure 6, these are the 5 green circles to the left of the red dashed vertical line indicating −4 um. These five eyes had ΔG values that ranged from −4.5 to −9.5 um. Based on the post hoc examination of the b-scans, segmentation errors were the primary cause of the FP classification in three of these eyes (Table 2). Figure 5A shows the circular b-scans and the cpRNFL plots for one of these eyes, ID122. The horizontal, double-ended white and black arrows indicate two regions with obvious segmentation errors, and the red arrows indicate individual locations for illustration. The segmentation errors such as shown here are relatively subtle, but collectively can have a major impact when the ΔG criterion is only a few microns, −4 um for the criterion used here. We can estimate the impact of these errors by obtaining the area between the curves in the lower panels of Figure 5A. For example, in this eye the segmentation error in the region of the red rectangle, which is shown enlarged in panel B, contributes −2 um to the ΔG value of −6.3 um.

**Table 2.** Factors Contributing to the Five FP Eyes for a ΔG Criterion of −4 µm

FPs (N = 5)						
ID	P or NP	ΔG	Local	Segmentation	Alignment	Other
142	NP	−9.5	–	–	–	1-clipping
144	NP	−8.5	–	–	–	1-clipping
122	NP	−6.3	–	1	–	–
52	NP	−5.4	–	1	–	–
4	NP	−4.5	–	1	–	–

P, Progressing; NP, Not Progressing.

**Figure 7.**
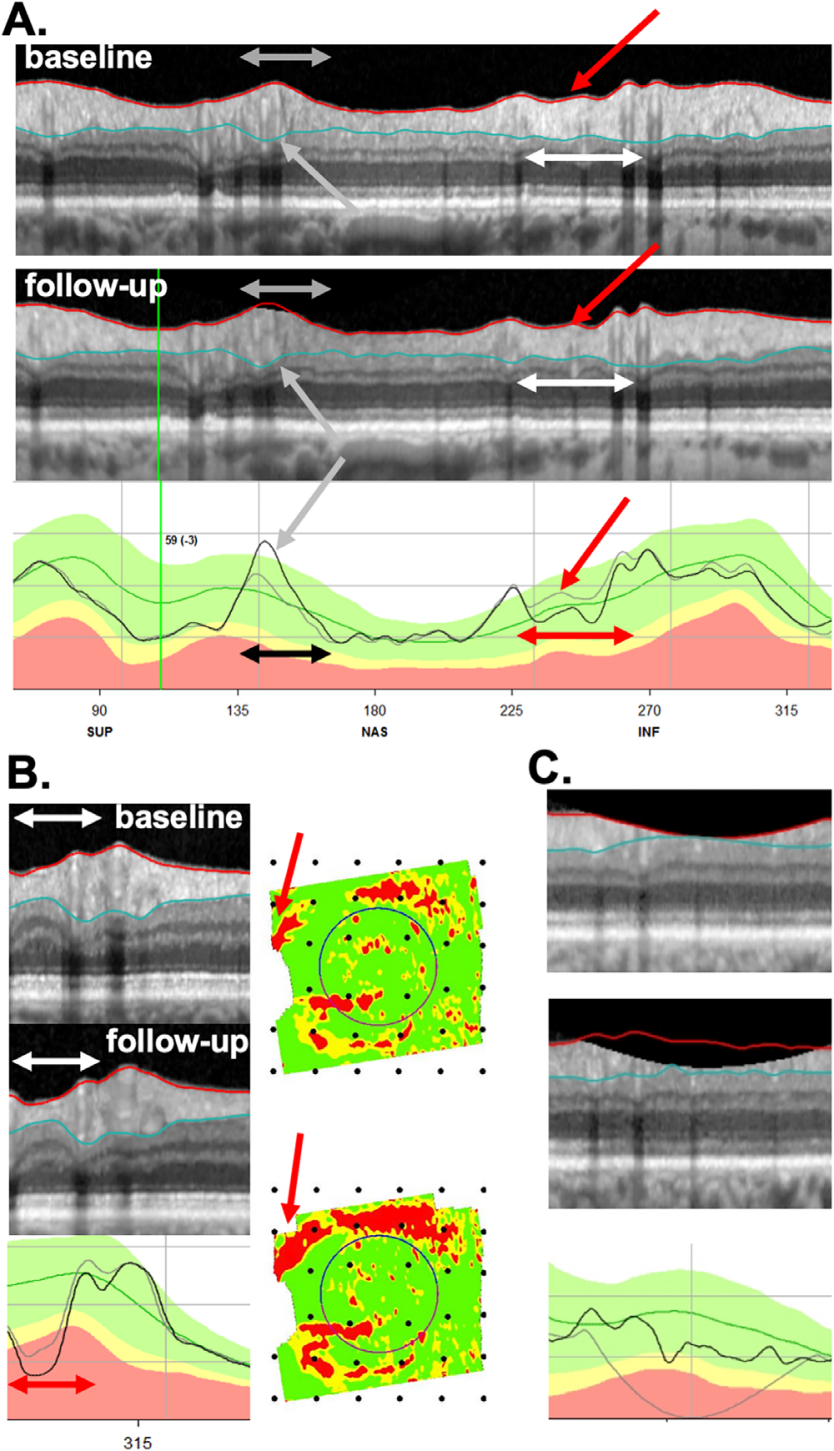
(A) An example of an FN eye (ID131), that is, a P eye that did not progress according to ΔG (−0.3 um). The *red arrow* points at the local region of progression, and the *double-ended white and red arrows* indicate the width of the local defect. A segmentation error (*gray arrows*) was associated with an increase in the ΔG value, while The *double-ended black arrow* indicates the width of the segmentation error. (B) Another example of an FN eye (ID132). The *double-ended white and red arrows* (left column) indicate the width of a local defect that has progressed. The *red arrows* (right column) point to corresponding locations on the RNFL probability plot, which is in field view. (C) A third FN (ID60) with a positive ΔG value of 3.0 um, that had a contribution from a segmentation error associated with clipping.

In the other two eyes, the primary cause was “clipping” of the b-scan due to a scanning error. Figure 5C shows the b-scans and cpRNFL thickness plots for one of these eyes, with the region “clipped” on follow-up indicated by the white and black arrows. This clipping error accounted for the large ΔG value of −9.5 um for this eye. This is caused by an error in the acquisition of the scan. In particular, if the OCT device is too close to the patient's eye, then part of the scan is inverted and subsequently “clipped out” from the analysis. Although clipping was a major factor for two (40%) of the five FP eyes, it was a factor in only one (1.2%) of the remaining 86 eyes classified as P or NP (see Fig. 7C). For comparison, segmentation was a factor in 38 (44.2%) of these 86 eyes.

*Analysis for FN:* For the −4 um ΔG criterion, 16 (53%) of the 30 P eyes were FN (misses). Based on the post hoc examination of the b-scans, the most common cause for an FN was that the defect was “local” (Table 3). (Note: the * indicates the defect was slightly wider than 45°.) In fact, local damage was a factor (primary or secondary) in all 16 FNs. Local defects by themselves can be missed by the ΔG metric, even in scans without segmentation errors. Consider the local defects in Figure 7A for one P eye (ID131). The red arrows point to local regions of cpRNFL loss, and the horizontal white and red arrows the approximate width of the local defect. We estimated the total loss in cpRNFL within the local defects by calculating the area between the gray and black curves in the lower panels. If segmentation were perfect throughout 360° of the scans, the local defect would produce ΔG values of −1.9 um; however, segmentation errors are reasonably common, and errors in other parts of the scan can either increase or decrease the ΔG value. The actual changes for this eye was −0.3 as segmentation errors (gray and black arrows in Fig. 7A) brought ΔG closer to zero. Figure 7B (ID132) shows another example in which progression of the local defect, seen on the cpRNFL plot (red horizontal arrows), is confirmed on the b-scan (white horizontal arrows) and RNFL probability maps (red arrows). In this case, this local defect alone would yield a ΔG value of −1.1 um; the measured ΔG value was −3.6 um. Finally, one P eye (ID60) with the positive ΔG value, +3 um, had a contribution from a segmentation error associated with clipping (Fig. 7C).

**Table 3.** Factors Contributing to the 16 FN Eyes for a ΔG Criterion of −4 µm

FNs (misses) N = 16						
ID	P or NP	ΔG	Local	Segmentation	Alignment	Other
6	P	−3.96	2	1	–	–
132	P	−3.6	1	2	–	–
81	P	−3.0	1*	–	–	–
114	P	−2.6	1	2	–	–
40	P	−2.3	1	–	–	–
31	P	−1.8	1	2	–	–
95	P	−1.8	1	–	–	–
104	P	−1.7	2	1	–	–
7	P	−1.5	1*	1	–	–
35	P	−1.4	2	1	–	–
79	P	−1.3	1	2	–	–
12	P	−0.9	1	–	–	–
98	P	−0.9	1	–	–	–
131	P	−0.3	1	2	–	–
94	P	0.6	1	2	–	–
60	P	3.0	1*	2	–	3-clipping

P, Progressing; NP, Not Progressing.

### Post Hoc Analysis of TP and TN

**Figure 8.**
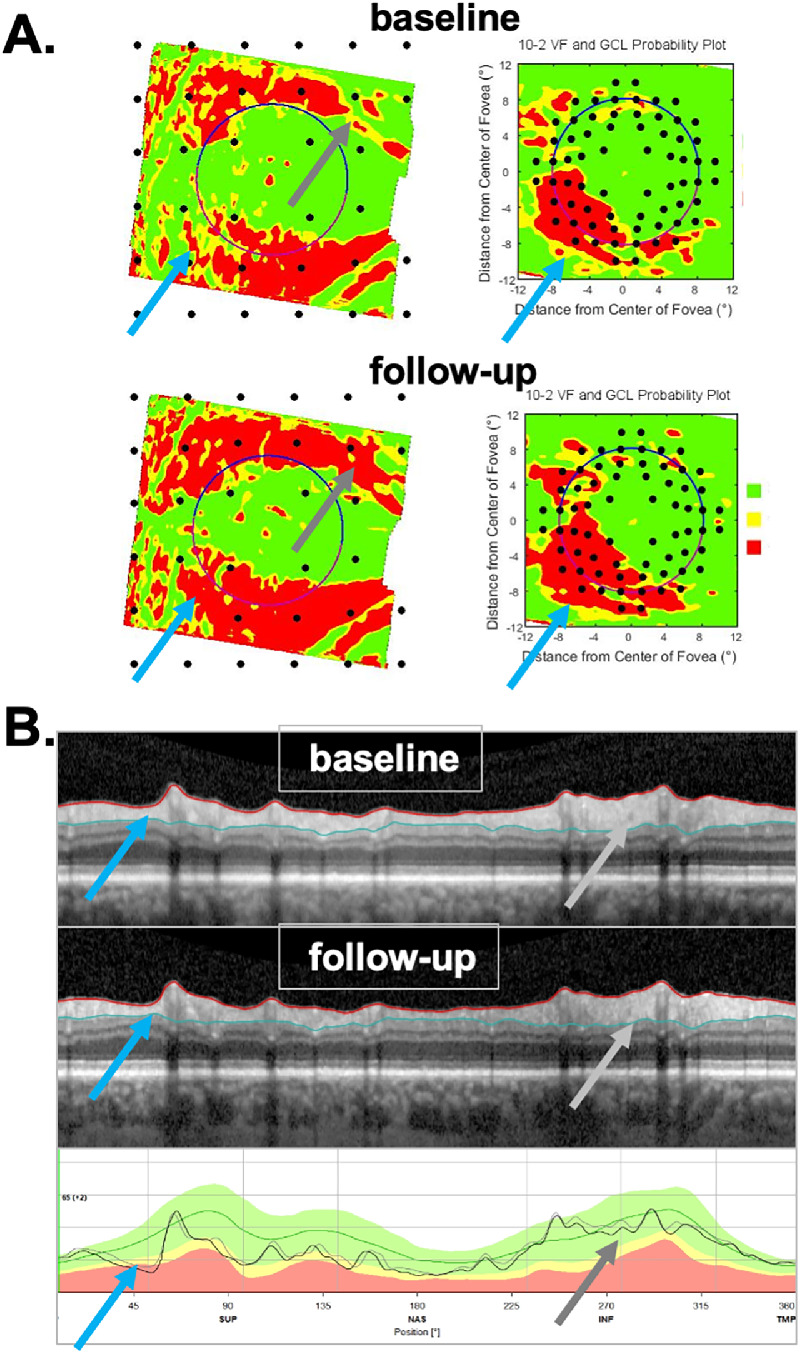
Example of an eye (ID75) that was a TP as confirmed by the probability maps in (A) and reflected in the widespread progression seen on the b-scan and thickness map in (B). The *arrows* point to regions in (A) and (B) associated with the same defects in the superior retina/disc (*light blue*) and in the inferior retina/disc (*gray*).

Of the 30 eyes categorized as P by the RS, 14 eyes were “true positives” (TP) based on the criterion level of ΔG (−4 um) (see Supplementary Table S1). The ΔG values of these eyes ranged from −4.1 to −12.1 um. As expected, all showed progression on the b-scans and cpRNFL plots. In fact, progression was largely responsible for the negative ΔG value. This progression included local defects in five eyes. However, it is worth noting that the progression included large portions (from approximately 270°–360°) of the scan in most of these eyes. Figure 8 (ID75) shows an example in which progression, which was confirmed by the probability maps in the left panels, involved a small decrease in cpRNFL thickness over much of the b-scan. Although these 14 eyes were TP based on ΔG (−4 um), this did not mean they were immune to errors. Scaling errors were evident in 3 of the 14 eyes, and segmentation errors in 4 (Supplementary Table S1).

Of the 61 eyes categorized as NP by the RS, 56 eyes were “true negatives” (TN) based on the ΔG criterion of −4 um. The ΔG values for these eyes ranged from −3.6 to 5.3 um (see Supplementary Table S2). Although these were TN eyes, the ΔG values were still influenced by segmentation errors in 14 (25.0%) eyes, scaling in 5 (8.9%) eyes, and alignment in 2 (3.8%) eyes. Note: as expected, alignment errors did not appear to play a major role, as these were the only eyes in the 91-eye P or NP group showing an alignment error.

### Post Hoc Analysis of Eyes with Extreme ΔG

**Figure 9.**
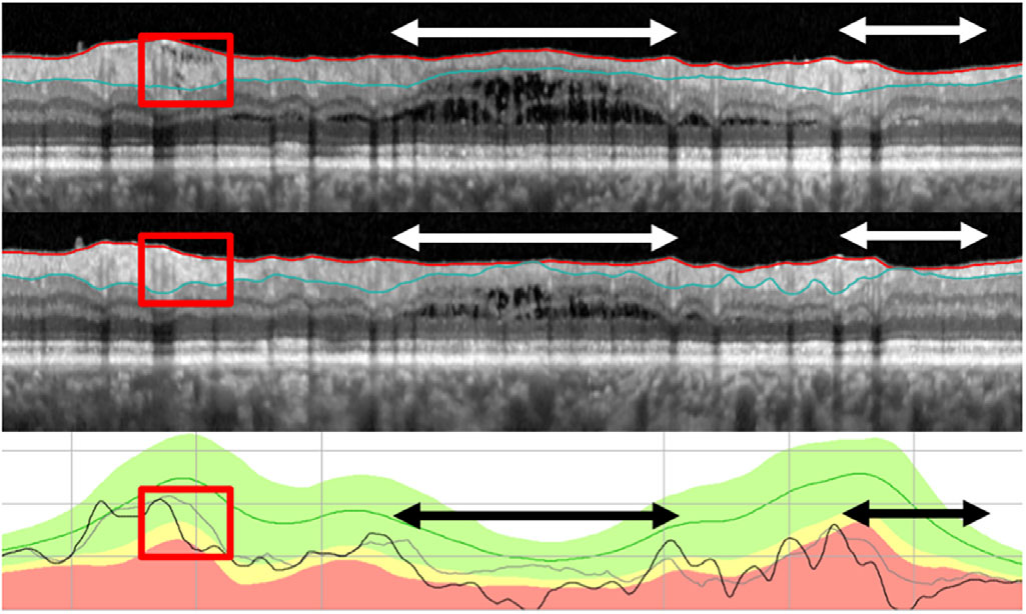
An example of an eye (ID137) that had an extreme ΔG value (−17.5 um) but was “uncertain” based on the RS. The regions with the *white and black double-ended arrows* have segmentation errors and the region within the *red rectangles* shows a cpRNFL that decreased in thickness due to an improvement of the schisis secondary to an ERM.

In addition to examining the b-scans of eyes classified as P or NP based on the RS, we also looked at the outliers among the 49 uncertain eyes (i.e., neither P nor NP). These six eyes are indicated with the gray arrows in Figure 6 and the results shown in Table 4. There were four eyes with the extreme positive ΔG values, which ranged between 21.2 and 6.6 um. All four had segmentation problems, whereas the three eyes with the largest ΔG values (11.7, 21.1, 21.2) had clipping combined with segmentation errors. The two eyes with the extreme negative ΔG values (−12.6 and −17.6) also had large segmentation errors. Figure 9 shows the b-scans for one of these eyes (ID137). The regions with the white and black horizontal lines and arrows indicate regions with segmentation errors. However, note that a partial improvement in schisis secondary to an epiretinal membrane (ERM) also contributed, as indicated by the region within the red rectangles in Figure 9.

**Table 4.** Factors Contributing to the Extreme Positive and Negative ΔG Values

ID	P or NP	ΔG	Local	Segmentation	Alignment	Other
62	–	21.2	N/A	1	–	clipping
143	–	21.1	N/A	1	–	clipping
1	–	11.7	N/A	1	–	clipping
101	–	6.6	N/A	1	–	–
57	–	−12.6	–	1	2-minor	–
137	–	−17.6	–	1	–	schisis

P, Progressing; NP, Not Progressing; N/A not available.

### Post Hoc Analysis of Eyes with High Myopia

Of the 25 study eyes with a refractive error less than −6 D, 10 were classified as NP and 4 as P. There was no relationship between the refractive error and the number of eyes classified as NP or P. In particular, these 14 eyes represented 15.4% of the 91 eyes classified as NP or P, and the 25 eyes with high myopia were 16.7% of the total 150 eyes. Only one of the NP eyes was found to be a FP, whereas there were no FNs. The only FP suffered from segmentation errors. In addition, three more high myopic eyes (one NP, two uncertain) had segmentation issues, but these did not significantly affect ΔG.

## Discussion

The change in the cpRNFL thickness, the ΔG metric, is used by some clinicians to track progression and is displayed on various commercial OCT reports. However, recent work^5^^,^^7^ has argued that this metric does not perform well, and our results here are in agreement. There is also evidence that the cpRNFL thickness measured from circle b-scans, on which the ΔG metric is based, is affected by segmentation and alignment errors^14^^–^^18^ and that reports based on this metric can miss local defects.^19^^–^^21^ Our purpose here was to provide evidence that links these findings. That is, we tested the hypothesis that local defects, segmentation errors, and alignment are major contributing factors to the relatively poor performance of the commonly used method to detect progression, that is, the ΔG metric. In particular, we predicted that local defects and segmentation errors would negatively impact performance of the G metric (ΔG), but that alignment errors would be minimized by the eye tracking used in scan acquisition in this study.

To test our hypothesis, we used an RS to identify 91 of the 150 eyes as P or NP. Based on this RS, and the best ΔG criterion (−4 um), there were 5 FP and 16 FN. An examination of the circumpapillary b-scans of these eyes indicated that, as predicted, alignment errors were not a significant factor, and, as predicted, local defects and segmentation errors negatively impacted performance of the ΔG metric. For example, the FN (misses) of a group of P eyes tended to have local defects, often accompanied by segmentation errors that made ΔG less likely to detect change. Blood vessel locations were particularly vulnerable to segmentation errors. In addition, ocular conditions such as ERM and schisis, scanning artifacts such as clipping, and small changes in image scaling/magnification can also contribute. There are several aspects of these findings worth emphasizing.

### Progression of Local Defects will be Missed by ΔG, and can be Obscured by Segmentation Errors

Local defects may contribute relatively little to ΔG values. Thus it will be difficult to detect changes in these defects using ΔG, especially as they can be associated with other errors, such as segmentation errors. For example, the local defect in Figure 7B only contributes −1.1 um to ΔG. Past work has emphasized that ΔG will miss local progression.^19^^–^^21^ Although this is true, it is probably more accurate to say that to detect progression using a ΔG cutoff, progression needs to be relatively widespread. Thus it is not surprising that we found that all TP detected by ΔG showed relatively widespread regions of progression (Supplementary Table S1), and further that the FN (misses) tended to be those eyes with relatively local defects (Table 3).

### The Region of Uncertainty or why Methods Such as the “Rule of 5” Fail

There is no ΔG cutoff that will result in high sensitivity for detecting progression with a high specificity. For example, consider the range of ΔG values between −4 and +3 um (the red dotted rectangle in Fig. 6). We call this the “uncertainty range” as it includes ΔG values associated with eyes that are NP (n = 54), with eyes that are P (n = 14), and with eyes for which we are uncertain (n = 40). Further, given the problems identified earlier, adjusting the fixed ΔG criterion level, or using a regression technique to set ΔG, will not allow the clinician to confidently use ΔG by itself for clinical judgments.

### Apparent Change in Overall Retinal Thickness

The alignment of b-scan images from the two test days had relatively little effect on ΔG values, presumably due to eye tracking employed by the instrument used in this study. In fact, the excellent alignment between scan dates allowed us to identify another factor, an “apparent change of retinal thickness” (aΔRT). We called it an “apparent” change as it is not clear what is causing these changes, and whether the causes are physiological or nonphysiological in nature. By nonphysiological we mean, for example, subtle differences in the patient's orientation relative to the scanning instrument and plane of scanning, and by physiological changes we mean factors such as IOP, which some studies have found affect retinal and choroidal thickness measures.^22^^–^^24^ However, our data show weak support at best for IOP involvement. Of the eight eyes identified with aΔRT changes, only two had IOP changes greater than 3 mm Hg between the test dates; one changed from 17 to 26 mm Hg and the other from 12 to 26 mmHg. Although both eyes were among the five of eight with reduced retinal thickness (i.e., a negative aΔRT) between test dates, there was no clear relation between change of IOP and aΔRT in the other six eyes.

In any case, the aΔRT values are small, on the order of at most 5% of retinal thickness. In fact, in our study, only eight eyes had an aΔRT large enough to be reliably identified, and they did not negatively impact the accuracy as three were TPs and five were TNs. Further, it is likely that these aΔRT exist in the data from all commercial instruments. However, they will not be noticed unless the images from different days are carefully aligned, as is the case in the current study in which the instrument employed eye tracking. In addition, because these aΔRT changes are small, they are not likely to affect a qualitative analysis of scan images. However, they are not insignificant when quantitative criteria such as the rule of 5 is employed. For example, if 80 um of RNFL remains, a 5% change is 4 um, or 80% of change needed for “progression.”

### Clinical Relevance

The primary implications of this study are the following: first, the clinician should not make judgments about progression based strictly on global metrics such as ΔG. This is not new to clinicians as similar arguments have been made for perimetry in the past.^25^ Second, and most importantly, before making a judgement about progression, clinicians should examine the circumpapillary b-scan images to look for signs of glaucomatous damage and to confirm that there are no artifacts (e.g., clipping), other pathologies (e.g., ERMs), or segmentation errors affecting the segmentation of the cpRNFL.^14^^–^^16^ Liu et al.^17^ emphasized this point in 2015. If the cpRNFL thickness measures can be trusted, then the cpRNFL thickness curves, such as in panel 4 of Figures 1B and 3B, can be compared. If the curve from time 2 falls essentially on top of the curve for time 1, as in Figure 1B, the clinician can be reasonably certain that little or no progression has taken place. If the curve from time 2 falls below the curve for time 1 either in a local region or in a more widespread fashion, this suggests possible progression, which needs to be confirmed. Third, to confirm either progression or the lack thereof, the clinician should make sure the appearance of the cpRNFL on the b-scan image and the RNFL and GCL deviation/probability plots (panels 5 and 6 of Figs. 2 and 4) are consistent. Note: it is important to remember that the analysis of cpRNFL alone can miss macular damage in early glaucoma.^26^

### Limitations

There are two important limitations to be considered. First, the eyes in this study did not include eyes with advanced glaucoma. However, it is generally believed that OCT has limited use in this group. Although we agree that this is true if one depends on metrics such as G, we have presented evidence that the approach that depends on scrutinizing circular b-scans and probability plots, described earlier, can be used to identify glaucomatous damage in many eyes with advanced glaucoma.^27^

Second, some will argue we are being unfair as we did not discard eyes with obvious problems, such as “clipping,” and that we did not correct segmentation, as the manufacturer suggested. We chose not to do either as we wanted to be close to common clinical practice. In particular, most clinicians are not looking at the scans to identify these errors and, in our experience, even fewer are correcting segmentation errors. In any case, of the 150 eyes, the scans of 8 eyes had clipping errors, whereas 11 had aΔRT errors, and 10 had alignment errors, although in most cases not large enough to noticeably affect ΔG. Although clipping errors are easy to identify, aΔRT and alignment errors are not. Nevertheless, if these 29 eyes were removed from the analysis, the remaining 121 eyes would still have the problems with segmentation and local defects identified earlier. Concerning segmentation, even if the clinician had the time or technical help to make these corrections, it would be very difficult to correct subtle segmentation errors such as those as seen in Figure 5A, particularly around blood vessels. However, even if it were possible to adequately correct segmentation, examinations of b-scans and probability maps, as described earlier, will detect changes missed with ΔG. The only possible exception is subtle wide-spread damage, and even this needs to be confirmed as automated segmentation shows better repeatability than manual segmentation.^28^

## Conclusions

Global measures such as changes in average cpRNFL thickness will miss progression of glaucoma. As it is an average, it can also miss local defects, or in other words be more likely to detect widespread thinning. Further, global cpRNFL thickness is prone to FP and FN mistakes due primarily to segmentation errors when care is taken to align scans. More robust approaches are needed that do not rely on metrics and instead focus on the agreement among the b-scans, thickness map, and probability/deviation plots.

## Supplementary Material

Supplement 1

Supplement 2

Supplement 3
