# Supplementary figures and images for: Reasons why OCT Global Circumpapillary Retinal Nerve Fiber Layer Thickness is a Poor Measure of Glaucomatous Progression
Source: Transl Vis Sci Technol. 2020 Oct 19;9(11):22. doi: 10.1167/tvst.9.11.22 (PMC7585398; doi:10.1167/tvst.9.11.22)

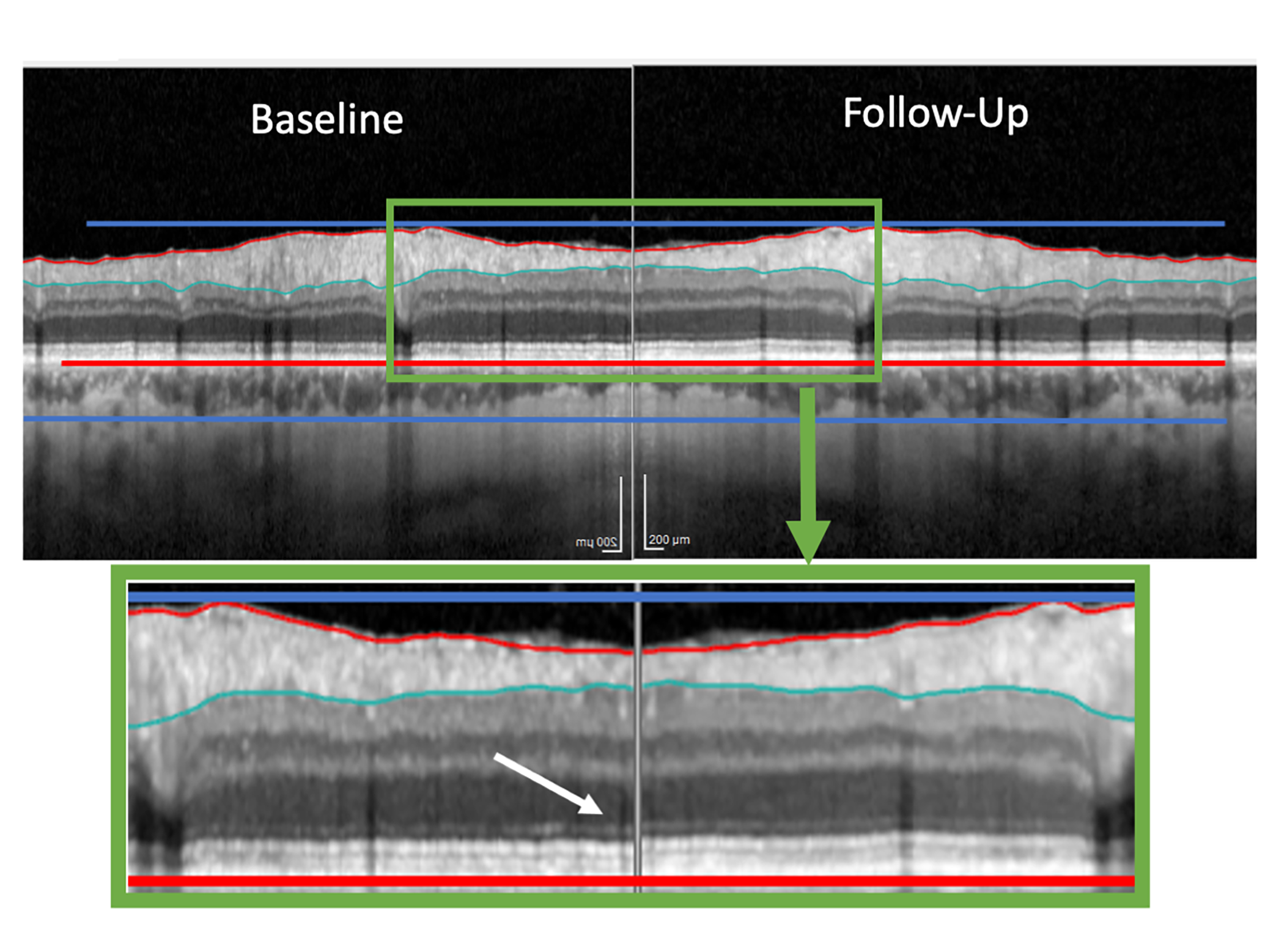

Supplement: Supplement 1 [file tvst-9-11-22_s001.tif]

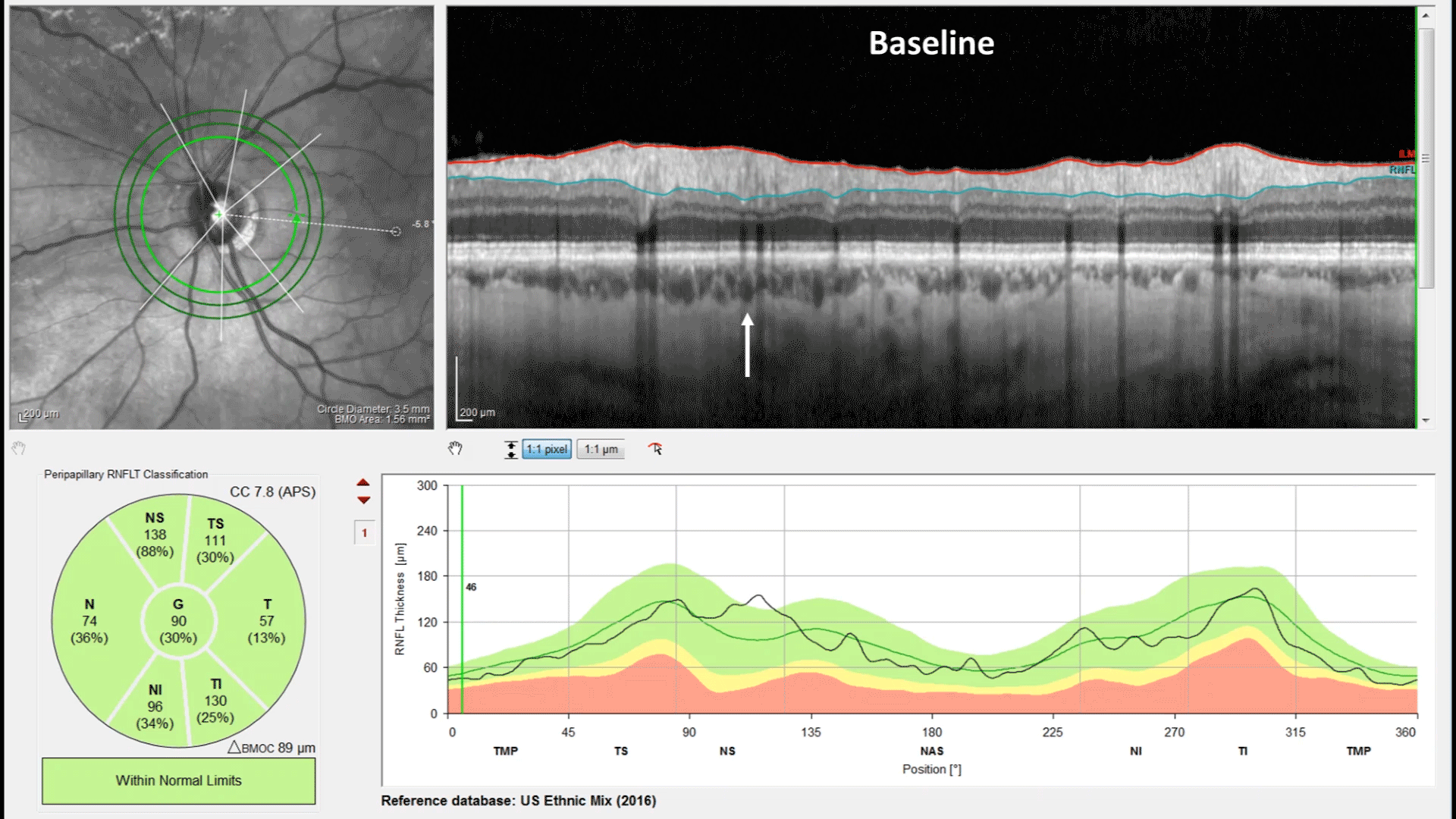

Supplement: Supplement 2 [file tvst-9-11-22_s002.gif]
