# Supplementary material for: Reasons why OCT Global Circumpapillary Retinal Nerve Fiber Layer Thickness is a Poor Measure of Glaucomatous Progression
Source: Transl Vis Sci Technol. 2020 Oct 19;9(11):22. doi: 10.1167/tvst.9.11.22 (PMC7585398; doi:10.1167/tvst.9.11.22)
Supplement: Supplement 3 [file tvst-9-11-22_s003.pdf]

Table S1. Characteristics of the b-scans of the 14 true positive (TP) eyes for a ΔG criterion of -4 um.

True Positives (n=14)						
**ID**	**P or NP**	**DG**	**Local**	**Segmentation**	**Alignment**	**Other**
118	P	-12.1	1-WS	2		
48	P	-12.1	1-WS			
39	P	-9.9	1-WS			
47	P	-8.3	1- WS+L			
146	P	-7.7	1-WS			2-scaling
161	P	-7.6	1-WS	2		3-scaling
100	P	-7.3	1-WS	2		
149	P	-7.1	1-WS			2-scaling
147	P	-5.6	1-WS+L			
75	P	-5.4	1-WS			
11	P	-5.3	1-WS+L			
150	P	-5	1-WS	2		
93	P	-4.8	1-WS+L			
53	P	-4.1	1-WS+L			

Table S2. Characteristics of the b-scans of the 56 true negative (TN) eyes for a ΔG criterion of -4 um.

True Negatives (n=56)					
**ID**	**P or NP**	**DG**	**Segmentation**	**Alignment**	**Other**
5	NP	-3.6	1	2	
56	NP	-3.0			
22	NP	-3.0	1		scaling
51	NP	-2.9			
120	NP	-2.9			
138	NP	-2.6	1		schisis
85	NP	-2.6	1	2	
3	NP	-2.6			scaling
30	NP	-2.5			
140	NP	-2.5			
9	NP	-2.4			
126	NP	-2.4	1		
83	NP	-2.1			
36	NP	-2.0			

61	NP	-2.0			
63	NP	-1.9	1		
111	NP	-1.8			scaling
67	NP	-1.8	1		
135	NP	-1.6			
10	NP	-1.4			
125	NP	-1.4	1		
54	NP	-1.3			
29	NP	-1.2			
19	NP	-1.0			
59	NP	-0.8			
117	NP	-0.8			
156	NP	-0.8			
23	NP	-0.7			
110	NP	-0.7			
55	NP	-0.6			
106	NP	-0.6			
20	NP	-0.5			

91	NP	-0.5			
113	NP	-0.4			
88	NP	-0.3	1		
99	NP	-0.3	1		
92	NP	-0.2			
103	NP	0.0			
116	NP	0.0			
148	NP	0.0			
70	NP	0.1	1		
28	NP	0.3			
82	NP	0.4			
157	NP	0.4			
112	NP	0.4			
41	NP	0.5			
128	NP	0.6			
139	NP	0.6			
119	NP	0.7			ERM
145	NP	0.9			scaling

46	NP	1.3			
107	NP	1.3	1		
65	NP	1.4			
25	NP	2.0			
89	NP	4.8	1		scaling
66	NP	5.3			
